# ATAD2 as a Cancer Target: Insights into Its Structure, Functions, Mechanisms, and Drug Development

**DOI:** 10.3390/cancers17203337

**Published:** 2025-10-16

**Authors:** Tanya Garain, Prateek Rai, Wei Li, Souvik Banerjee

**Affiliations:** 1 Molecular Biosciences, Middle Tennessee State University, Murfreesboro, TN 37130, USA; tg5s@mtmail.mtsu.edu (T.G.); pr3x@mtmail.mtsu.edu (P.R.); 2 Department of Chemistry, Middle Tennessee State University, Murfreesboro, TN 37130, USA; 3 Department of Pharmaceutical Sciences, College of Pharmacy, University of Tennessee Health Science Center, Memphis, TN 38163, USA; wli@uthsc.edu

**Keywords:** ATAD2, ATPases, bromodomain, cancer biology, drug resistance, drug discovery, molecular modeling, pharmacology

## Abstract

**Simple Summary:**

Cancer remains a major cause of death worldwide, driven by numerous molecular pathways that support tumor growth, survival, and progression. The search for new therapeutic targets has identified several promising proteins that act through distinct mechanisms. One such target is ATPase family AAA domain-containing protein 2 (ATAD2), a protein that regulates how genes are organized and expressed in cells. Elevated ATAD2 levels are found in many cancers, where it promotes tumor proliferation, survival, and metastasis, and emerging evidence suggests it may contribute to resistance against existing therapies. This review provides a comprehensive overview of ATAD2, including its structure, biological functions, and role in cancer progression. It also highlights strategies being explored to inhibit ATAD2, particularly small-molecule approaches, and presents computational insights that enhance our understanding of its interactions. By consolidating these findings, the review aims to guide future research toward effective ATAD2-targeted therapies.

**Abstract:**

ATPase family AAA domain-containing protein 2 (ATAD2) has been recognized as a key oncogene that regulates chromatin remodeling, transcription, and cancer progression. As a member of the bromodomain (BRD) family, ATAD2 plays a crucial role in epigenetic modifications and is associated with multiple malignancies. Despite being considered an undruggable target in the past, crystallography and computational modeling have significantly accelerated ATAD2 drug discovery and development. This review provides a comprehensive overview of the structural features, functional roles, and biological significance of ATAD2, particularly in the context of cancer. We present an in-depth overview of different molecular strategies reported in the literature to suppress ATAD2 expression, including genetic and pharmacological approaches, and discuss their mechanistic and therapeutic implications. Particular emphasis is given to recent efforts in developing small-molecule inhibitors, detailing their binding interactions, therapeutic potential, and challenges in clinical translation. In addition, we performed alanine scanning calculations on molecular dynamics (MD)-simulated trajectories derived from protein–ligand complexes based on X-ray co-crystal structures containing three distinct ligands with different binding modes. This analysis provided critical insights into the binding interface of BRD-ATAD2, enhancing our understanding of its ligand interactions. Furthermore, we examine the emerging roles of ATAD2 in mediating resistance to cancer therapies, underscoring its potential as a target for overcoming drug resistance. By integrating structural insights, mechanistic studies, drug discovery efforts, and the challenges of developing ATAD2-targeted cancer therapies, this review emphasizes the need for further research to optimize ATAD2 inhibition strategies and explore its full therapeutic potential in oncology.

## 1. Introduction

All organisms contain members of the large and diverse AAA+ superfamily of proteins known as AAA ATPases [1,2]. These proteins have a significantly conserved ATP-binding N-terminal domain comprising about 200–250 amino acids. They typically oligomerize into hexameric ring complexes, catalyzing the hydrolysis of ATP to ADP with the release of phosphate ions, converting chemical energy into mechanical force [3]. The chemical energy stored as a phosphate bond in ATP is used in various cellular processes such as protein synthesis and degradation, DNA repair and replication, maintaining genomic stability, chromatin remodeling, organelle biosynthesis, transport of metabolites and nutrients, etc. [4]. Extensive research has made it possible to identify more members of the ATPase family and their specific functions. Among them, ATPase family AAA domain-containing protein 2 (ATAD2), also known as AAA+ nuclear coregulator cancer-associated (ANCCA), has emerged as a significant target because of its important role in cancer [5,6,7,8,9,10].

ATAD2 belongs to the family IV bromodomain-containing (BRD) protein and is a cancer-testis antigen (CTA) localized to the nucleoplasm. It is located at chromosomal location 8q24.13 and has 31 exons. Under non-malignant circumstances, ATAD2 is mainly expressed in male germ cells, embryonic stem (ES) cells, and bone marrow. Its concentration is significantly low or undetectable in normal somatic cells. As a result, cells with extremely dynamic chromatin that experience significant remodeling express ATAD2 [11]. Initially, overexpression of AIB1, RAC3, and TRAM1 (known as ACTR) was found to promote proliferation in breast cancer (BC) and to confer anti-E2 resistance [12]. Zou et al. [13] discovered ATAD2 as an estrogen receptor α (ERα) coactivator in 2007 [13]. In their study, a gene expression microarray was conducted to study BC cells treated with E2 or the overexpression of ACTR to better understand its biological mechanism. An open reading frame (ORF) identified as pro2000 (later known as ATAD2), containing two AAA+ ATPase domains (AAA-D1 and AAA-D2) and a BRD, was shown to be upregulated in both array investigations. Later, it was reported that histone hyperacetylation on ER chromatin and the incorporation of cAMP-responsive element binding protein (CBP/p300) are controlled by ATAD2 binding and ATP hydrolysis [13]. Since then, many research groups have reported on the pleiotropic cancer-related functions of ATAD2 through a variety of signaling pathways, such as the steroid hormone signaling pathway, the retinoblastoma (Rb)/E2F-cMyc pathway, the AKT pathway, the hedgehog signaling pathway (HH), the hypoxia inducible factor 1α (HIF1α) signaling pathway, and the p53 and p38-MAPK-mediated apoptotic pathway in diverse cancer types [6,7,8,9,10,14]. The decade that followed saw an increase in the depth and breadth of ATAD2 research, from the upstream variables controlling ATAD2 protein production to the mechanisms underlying the onset and progression of malignancies in lung, ovary, breast, endometrial, liver, pancreas, stomach, bone, and colorectal [11]. In cancer cells, overexpressed ATAD2 interacts with a range of transcription factors and chromatin-modifying proteins to stimulate gene expression, which prevents apoptosis and promotes cell proliferation. In advanced malignant stages, ATAD2 is also linked with rapid mortality, short life expectancy, and disease recurrence [15].

## 2. ATAD2: A Member of the BRD Family of Proteins

**Figure 1:**
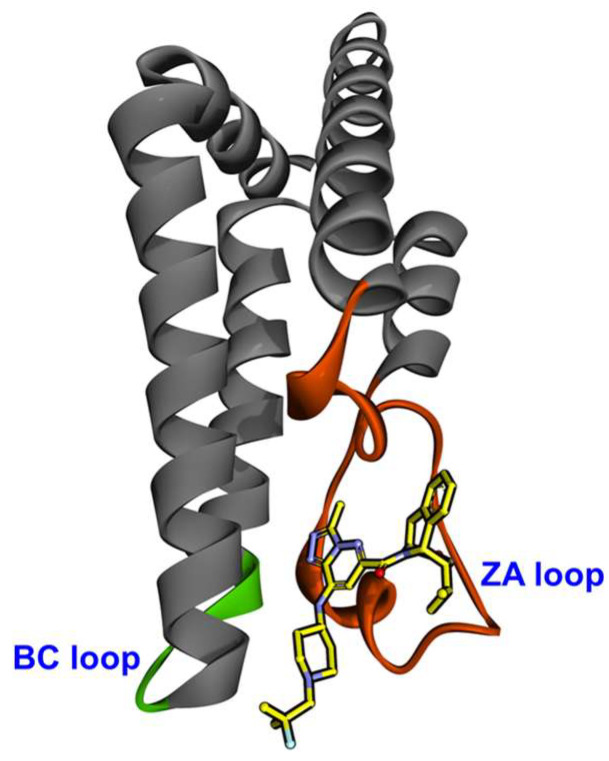
Crystal structure of the characteristic ZA and BC loops of the ATAD2-BRD (PDB ID: 7Q6T). The BRD comprises four bundles of α helices, with the ZA loop situated between αZ and αA helices and the BC loops situated between αB and αC helices. The ZA loop is highlighted in orange, and the BC loop is shown in green.

**Figure 2:**
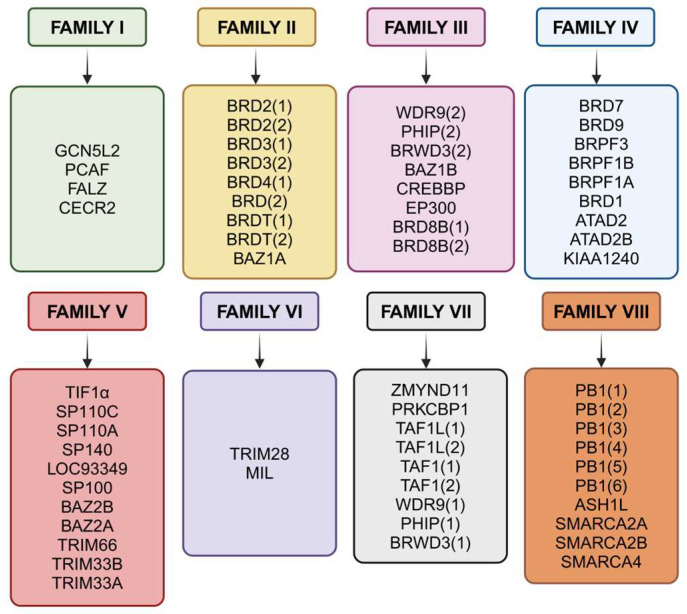
The eight subfamilies of human bromodomain-containing proteins (BRDs) are classified according to sequence and structural homology. ATAD2, the focus of this review, belongs to Family IV, together with BRD7, BRD9, and other BRPF family members.

BRDs are highly conserved evolutionary proteins primarily recognizing acetylated lysine residues on histones, although several BRDs can also identify acetylated non-histone proteins. It is well-established that BRDs control the transcriptional machinery via binding to acetylated lysine motifs, chromatin remodeling, and histone recognition [16]. The BRD comprises four bundles of α helices, with the ZA loop situated between αZ and αA helices and the BC loops situated between αB and αC helices (Figure 1) [4]. The co-crystal structures of BRDs linked to peptides containing acetyl-lysine indicate that the highly conserved residues of the loops form a hydrophobic pocket between them, which is where the acetylated lysine is found [17]. This includes an Asn (like Asn1064 of ATAD2-BRD) residue located at the center of the binding site, which forms a hydrogen bond with the acetyl-carbonyl oxygen atom of the acetylated lysine [18]. It has been shown that various BRD proteins are overexpressed and play a significant role in malignant tumors [19,20,21,22]. In the human genome, 46 distinct proteins collectively contain 61 bromodomains (BRDs), which are classified into eight subfamilies, based on protein sequence similarity (Figure 2); ATAD2 is a member of family IV BRD proteins [23]. Each of these proteins has a BRD that can identify and bind acetyl-lysine residues on histone tails; however, the histone marks each protein recognizes vary. It has recently been demonstrated that the ATAD2-BRD preferentially binds to the di-acetylated H4K5acK12ac mark present in histones that are newly synthesized after DNA replication [24]. Although ATAD2 has been widely studied, its homolog ATAD2B remains relatively underexplored [25]. With a 97% amino acid sequence homology for the AAA domain and a 74% amino acid sequence homology for the BRD, ATAD2 and ATAD2B are highly conserved and comparable [26].

## 3. Structural Insights and Functional Roles of ATAD2

**Figure 3:**
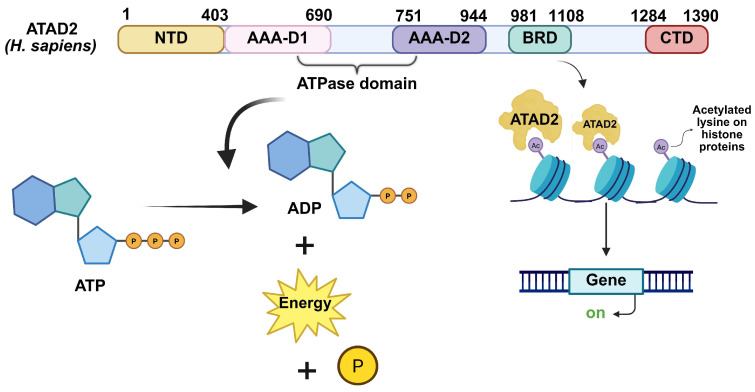
Domain organization and functional roles of human ATAD2. ATAD2 consists of an N-terminal acidic domain (NTD), two AAA+ ATPase domains (AAA-D1 and AAA-D2) forming the ATPase module, a bromodomain (BRD), and a C-terminal domain (CTD). The ATPase domains hydrolyze ATP to generate energy, supporting the role of ATAD2 as a chromatin remodeler. The BRD specifically recognizes acetylated lysine residues on histone proteins, facilitating ATAD2 recruitment to chromatin and promoting transcriptional activation of target genes. Ac stands for acetylated lysine; P stands for phosphate group liberated during the hydrolysis of ATP to ADP.

There are 1390 amino acids in ATAD2 (molecular weight 158.5 kDa), which are divided into the following domains: N-terminal domain (NTD) (residues: 1–403), bipartite domain; AAA-D1 (residues: 403–690), AAA-D2 (residues: 751–944), AAA+ ATPase domain (residues: 403–944), BRD (residues: 981–1108), and C-terminal domain (CTD) (residues: 1284–1390) (Figure 3). AAA-ATAD2 and BRD-ATAD2 are known to be highly conserved among all these domains and potential targets for therapy [27].

### 3.1. N-Terminal and C-Terminal Domains

The NTD (residues 1–403) of ATAD2 has a region of acidic amino acid residues, a characteristic observed across ATAD2 orthologs in different species. The acidic composition of the domain suggests its involvement in protein–protein interactions through electrostatic interactions. For instance, NTD is involved in the interaction of ATAD2 with androgen receptors and E2F1, a crucial transcription factor required for cell proliferation, DNA repair, and differentiation [28]. ATAD2-like proteins also share a CTD (residues 1264–1390) region located next to the BRD, which is not observed in any other class of protein [11,13,29,30].

### 3.2. AAA+ ATPase Domain

AAA-ATPases are characterized by their two different domains: AAA-D1 and AAA-D2. AAA-D1 is significantly homologous with other AAA+ ATPases, such as p97/VCP/Cdc48, Hsp104, and NSF, and is more conserved than AAA-D2 [13]. The AAA-ATAD2 component also includes conserved Walker A (GXXXXGKT/S) and B motifs (hhhhDE), sensors 1 and 2, and an Arg-finger [31]. AAA-ATAD2 regulates the binding between the enzyme and ATP, followed by the hydrolysis of ATP, and also facilitates the oligomerization of ATAD2 [32]. Cryogenic electron microscopy (cryo-EM) of Abo1, an ortholog of ATAD2, found in *Schizosaccharomyces pombe* [33], revealed six ATP-binding sites at the AAA-D1 interface. Walker A and Walker B motifs are not present in AAA-D2, and hence this domain has no catalytic function, resulting in no nucleotide detected at the interface. The hexameric structure of Abo1 is in a closed ring form in the apo-protein bound to ADP, and is in an open form when bound to ATP [27]. The AAA+ ATPase domain enables BRD-ATAD2 proteins to function by oligomerizing them. Zou et al. [13] used Walker A (K473T) and Walker B (E532Q) ATAD2 mutants to demonstrate that ATPase activity is required for ATAD2 to serve as a coactivator of ERα [13]. ATAD2 gains access to the replication sites of DNA and, using mutant ATAD2 (K473T and E532Q), disrupts the copying mechanism, highlighting the significance of the ATPase domain in the functions of ATAD2 [34].

### 3.3. Bromodomain (BRD)

BRD-ATAD2 (residues: 981–1108) has a structurally and evolutionarily conserved acetylated lysine (KAc) binding pocket (also known as “BRD fold”), analogous to other members of the BRD-IV family [35,36]. This motif consists of four alpha-helices (αZ, αA, αB, and αC) joined by ZA (αZ-αA) and BC (αB-αC) loops (Figure 1). The upper section of ZA and BC loops provides a hydrophobic pocket to recognize histone KAc sites. Crystal structures of BRD-ATAD2 in apo and peptide/ligand-bound states show significant conformational differences in ZA and BC loops, indicating uneven flexibility of the KAc cavity [37,38]. This flexibility of the KAc cavity impacts ATAD2’s ligand binding and function. Poncet-Montange et al. [38] reported that when the peptide is attached, two Pro residues (Pro1012 and Pro1028) act as hinges, allowing the ZA loop to move and the hydrophobic amino acid residues (Val1013 and Val1018) to migrate inside, sealing the cavity [38]. Later, Zhang et al. [39], through MD simulations of BRD-ATAD2, revealed the existence of two distinct conformations of the KAc pocket: a “closed” state (peptide-bound/holo) and an “open” state (peptide-free/apo) [39]. These studies imply that when histones or ligands engage, the pocket experiences substantial conformational changes (from open to closed). Like other BRD proteins, BRD-ATAD2 also uses a similar binding mechanism to bind to various acetylated histones such as H3K9ac, H3K14ac, H4K5ac, and H4K12ac [29].

Increased levels of AAA-ATAD2 mutants like K473T and E532Q, and BRD-ATAD2 mutants such as V1013A, Y1021A, Y1064A, and I1074A, impair acetylated histone binding and replication of DNA [34]. Wild-type ATAD2 competes with histone deacetylase 1 (HDAC1) for histone binding, and its BRD oligomerizes through the AAA+ ATPase domain of ATAD2 to perform its fundamental function [40]. The ATPase domain hydrolyzes ATP to facilitate BRD accessibility to histone-acetylated tails. Multiple BRDs increase protein capture efficiency, and in the multimeric state, interactions between the AAA+ ATPase domain and BRD-ATAD2 enhance binding to the histone H4 acetylation site [32].

## 4. ATAD2 and Its Major Biological Significance

### 4.1. Regulation of Transcriptional Machinery

Transcription is an extremely regulated biochemical process in which an RNA molecule is generated from a DNA template, converting genetic information stored in DNA into a corresponding RNA sequence. At the key transcriptional step, ATAD2 catalyzes the hydrolysis of ATP, and this enzymatic activity is critical for facilitating partial chromatin reorganization [41]. The actions of ATAD2 as an epigenetic reader during DNA transcription and the recognition and binding of histone acetylated lysine by its BRD were further shown by subsequent investigations [37]. ATAD2 is then recruited to the promoters of target genes, where it helps assemble protein complexes that activate transcription and modify histones to boost gene expression [42,43]. Recent observations indicate that ATAD2 is a pluripotent regulator of chromatin remodeling, promoting cell differentiation and proliferation [23,44,45]. The binding between ATAD2 and acetylated histone lysine increases access to chromatin, which is necessary for the highly expressed gene fraction to function effectively. According to the structural analysis of ATAD2, the AAA+ ATPase domain of BRD-ATAD2 can function as a molecular motor in precisely driving proteins to acetylated histones [13,46,47]. This mechanism involves the BRD reading the acetylated lysine on histones, the AAA+ ATPase domain promoting nucleosome eviction, and the acidic NTD of ATAD2 binding firmly but non-specifically to chromatin [32]. Since ATAD2 controls the transcription of a specific collection of genes that activate the proliferative activity of cancer cells through a variety of pathways, it functions as a crucial transcription factor or coactivator in malignancies [48].

### 4.2. Replication of DNA

**Figure 4:**
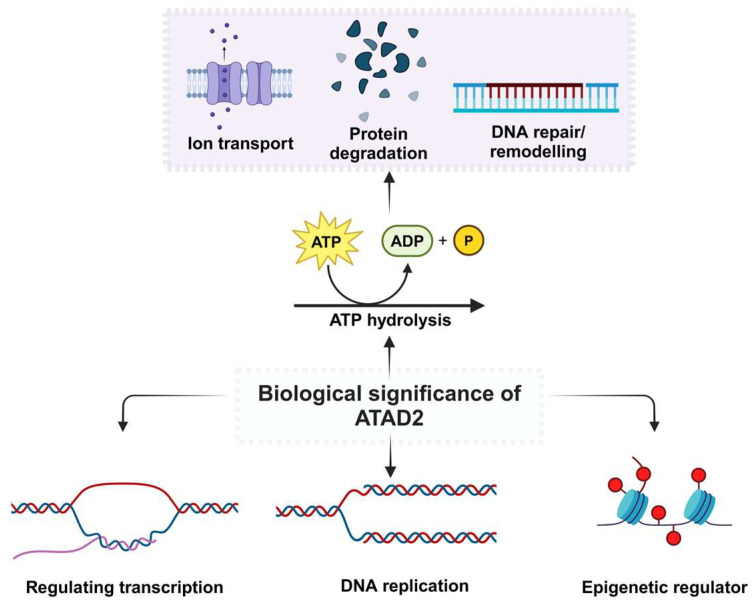
Involvement of ATAD2 in various biological processes. ATAD2 functions as an epigenetic decoder, transcription factor, and coactivator in various physiological activities, including DNA replication and transcriptional regulation, due to its BRD [47]. ATAD2’s ATPase domain, like others in the AAA+ superfamily, plays a crucial role in protein oligomerization and ATP binding and hydrolysis. ATAD2 breaks high-energy phosphate bonds and converts ATP into ADP and phosphate, releasing energy. The energy given supports essential cellular functions such as protein folding [51], intracellular transport [52], DNA repair, and ion transport [53].

DNA is the molecule that contains the genetic information necessary for an organism’s growth and operation. DNA replication is the primary mechanism underlying cell proliferation, which takes place in the S phase of the cell cycle [49]. Research has shown that ATAD2 is highly expressed in this phase and interacts with newly synthesized histones by getting recruited at the replication site during the replication initiation stage (Figure 4). This process involves the formation of diacetylation marks at K5 and K12 by histone H4 at the replication site [50]. During the recombination of replication-linked nucleosomes, the conserved Asn1064 residue in the hydrophobic pocket of the ATAD2 BRD directly interacts with diacetylated lysines [34].

## 5. ATAD2 in Cancer: Mechanistic Insights Across Signaling Pathways

**Figure 5:**
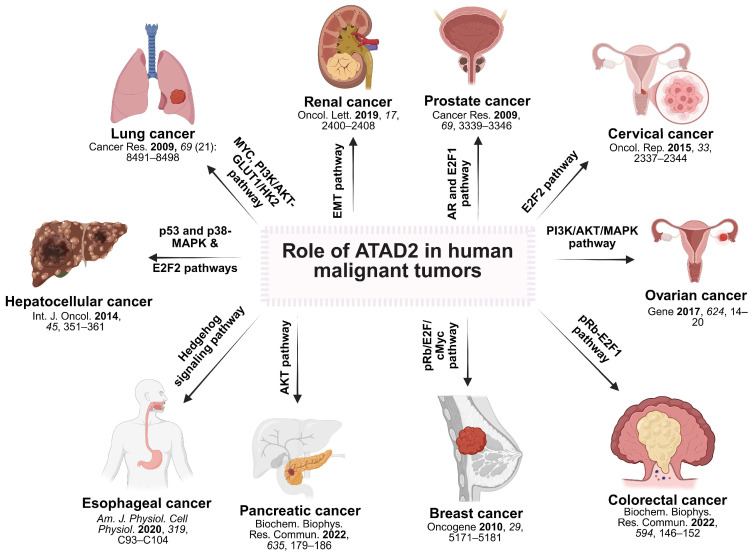
Role of ATAD2 in human malignant tumors. ATAD2 is implicated in diverse cancers, where it participates in or is modulated by multiple signaling pathways, including the Rb/E2F [40,54,55], AR/E2F1 [46], PI3K/AKT [56,57,58], MAPK [57,59], Hedgehog [60], and EMT pathways [61], among others [6,8,12,14]. While depicted separately here for clarity, these signaling routes are highly interconnected in cancer biology, often exhibiting forward–backward feedback loops. ATAD2 functions as a dynamic transcriptional co-regulator embedded within this complex network of pathways.

Cancer is an inherently intricate disease, driven by the simultaneous activation and suppression of multiple parallel signaling pathways. In this complex landscape, the precise biochemical functions of ATAD2 in cancer pathology remain an area of ongoing and evolving research. For instance, ATAD2 has been implicated in modulating the Rb/E2F axis that controls cell cycle progression, as well as oncogenic pathways such as PI3K/AKT and MAPK signaling, highlighting its potential to influence several regulatory networks that adapt during tumor progression. The several cancer pathways ATAD2 regulates are explained in detail in this section, along with the molecular underpinnings that make ATAD2 a viable target in cancer. The significance of ATAD2 in various cancers through distinct signaling pathways is also summarized in Figure 5 [11,54]. It is important to note, however, that these pathways do not operate in isolation. In cancer biology, extensive crosstalk, overlapping nodes, and forward–backward feedback loops are common, and ATAD2 functions as part of this interconnected network. Depending on the context, ATAD2 may act upstream as a transcriptional co-regulator (e.g., in the Rb/E2F or AR/E2F1 pathways) or be modulated by oncogenic cascades such as PI3K/AKT or MAPK.

### 5.1. Rb/E2F-cMyc Pathway: ATAD2 Stimulates the Proliferation of Cancer Cells

The Rb pathway controls cell division during the first phase of the cell cycle, G_1_ [62]. The Rb pocket family (Rb, p107, p130), D-type cyclins, cyclin-dependent protein kinases (CDK4, CDK6, CDKN), and the E2F transcription factor family (E2F1-8 and DP1-2) are the core components of Rb signaling, which is crucial for cell cycle control [29]. The expression of ATAD2 increases during the G_1_/S phase but decreases during the G_2_/M phase of the cell cycle [29,56]. The relationship between ATAD2 and Rb has been investigated in mouse embryonic fibroblast (MEF) cells that expressed high levels of ATAD2 mRNA and had the Rb gene removed [56]. In this study, it was found that in the absence of Rb protein, ATAD2 interacted with different E2F proteins, namely, E2F1, E2F2, and E2F3. The finding further indicates seven potential E2Fs binding sites in the ATAD2 regulatory region [29,56]. ATAD2-E2F complex then proceeds to bind the promoters and triggers the expression of genes such as cyclin A2, cyclin E1, cdk2, cdc6, and MCM7, consequently stimulating cell division [63]. Reiterating ATAD2’s involvement in the Rb/E2F pathway, a new study further demonstrates that in gastric cancer (GC) cells, suppression of ATAD2 delays the progression from G_1_ to S phase and lowers the production of cyclin D1, phosphorylated Rb (pRb), E2F1, and cyclin E. It has also been discovered that ATAD2 and E2F control the transcription of the proto-oncogene ACTR (AIB1/SRC-3/NCOA3) in BC cells [8]. There are E2F binding sites on the ACTR promoter, and ATAD2 is employed there along with E2F and ACTR to cause the proliferation of BC [12]. Moreover, ACTR engages with E2Fs and recruits itself to the target promoter’s E2F binding region to stimulate the production of E2F-targeted genes. For this reason, ATAD2 is essential for G_1_/S progression and the stimulation of cell-cycle proteins.

B-MYB (encoded by MYBL2) is a key oncogenic transcription factor that regulates genes involved in cell cycle progression, particularly the G_2_/M transition, often working together with E2F family members [64]. In triple-negative BC (TNBC), ATAD2 functions upstream as a transcriptional coactivator that promotes MYBL2 expression, thereby positioning B-MYB as a downstream effector of ATAD2 [65]. Inhibition of ATAD2 in TNBC cells leads to marked downregulation of B-MYB as well as genes essential for DNA replication and mitotic progression. Mechanistically, ATAD2 binds to the regulatory regions of several cell cycle–related genes, including Top2A, CDC2, cyclin A2, DLGAP5, Bub1, MCM10, KIF4A, KIF11, and KIF15, thereby sustaining proliferative capacity [64,65]. Beyond TNBC, ATAD2 deletion in colorectal cancer (CRC) cells reduces expression of cyclin D1, pRb, E2F1, and cyclin E, suggesting that the ATAD2–B-MYB axis is a conserved driver of oncogenic cell cycle control across multiple tumor types [66]. Myc, another important oncogene in the Rb-E2F pathway, activates E2F and proteins involved in the cell-cycle checkpoint, as a consequence to growth or mitogenic stimuli [67]. ATAD2 functions as a Myc cofactor, activating it to target genes like NUC, NOL1, GPAT, CCNDD2, HSP60, CAD, CDC25A, CDK4, AIM1, and CCNE1, subsequently stimulating the growth of osteosarcoma (OS) and lung fibroblasts [56]. Further, ATAD2 exhibits the highest synergy with Myc signaling in glioblastoma (GBM), breast, and ovarian malignancies among the genes upregulated in the 8q24 chromosomal region [7]. In hepatocellular carcinoma (HCC), ATAD2 suppression lowers Myc mRNA expression [59]. Furthermore, research on the gene expression of lung adenocarcinoma has shown a high correlation between ATAD2 and Myc, indicating that ATAD2 is a critical factor in Myc-regulated cell proliferation [9].

### 5.2. Involvement of ATAD2 in Steroid Hormone Signaling: Aiding the Survival and Multiplication of Cancerous Cells

ATAD2 actively mediates steroid hormones and their receptors, such as androgen and estrogen, which regulate gene expression, driving cellular proliferation and differentiation [68].

#### 5.2.1. Estrogen-Derived Cancer Is Induced by Dysregulated ATAD2

As mentioned previously, Zou et al. [13] have demonstrated that ATAD2 controls the signaling of ERa. In BC cells, E2 upregulates ATAD2, which is triggered by the employment of ACTR at the promoter region of ATAD2 [13]. Earlier, this same group had demonstrated that the interaction between ACTR and the ERα stimulates the growth of BC cells [12]. ATAD2 is recruited to the promoters of ERα-target cell cycle regulators, including cyclin D1, E2F1, and c-Myc, and interacts directly with both ERα and ATR. These factors drive proliferation: cyclin D1 activates CDK4/6 to promote the G_1_–S transition; E2F1 induces genes controlling DNA replication and S-phase entry; and c-Myc regulates transcriptional programs supporting cell growth and metabolism [69,70,71].

Mitotic kinesins (Kif4A, Kif15, Kif20A, and Kif23) play essential roles in spindle dynamics and cytokinesis [72]. They are also reported to increase in ER-positive BC cells because of enhanced ATAD2 expression independently and in response to E2. At the promoter location of these kinesins, estrogen triggers ATAD2 recruitment, which then facilitates the assemblage of E2F and MLL1, which is a histone H3K4 methyltransferase, resulting in E2-induced kinesin expression [6]. Overexpression of kinesins and ATAD2 was also found to be crucial for the development and viability of tamoxifen-resistant BC cells [6].

#### 5.2.2. Androgen-Dependent Oncogenesis Is Integrated by Aberrant Expression of ATAD2

ATAD2 is expressed in androgen-dependent and androgen-sensitive prostate cancer (PC) cells in response to androgen stimulation, and its levels are further elevated in androgen-independent and hormone-refractory PC cells. Functionally, ATAD2 acts as a coactivator of the androgen receptor (AR), enhancing the transcriptional activity of AR at target gene promoters such as IRS-2 and SNK1. Through this mechanism, ATAD2 overexpression induces the activation of AR-regulated genes that are critical for the survival and proliferation of both AR-positive and hormone-refractory PC cells [46].

Enhancer of zeste homolog 2 (EZH2), a histone methyltransferase and key component of the Polycomb repressive complex 2 (PRC2), is frequently overexpressed in prostate cancer and contributes to tumor progression. In prostate cancer cells, testosterone induces the expression of both EZH2 and ATAD2. Notably, inhibition of ATAD2 reduces EZH2 expression, indicating that EZH2 upregulation is mediated by ATAD2. Although EZH2 is a known E2F1 target gene, testosterone promotes ATAD2 recruitment to the EZH2 promoter, where ATAD2 functions as an E2F coactivator to facilitate RNA polymerase II and MLL1 loading. MLL1, in turn, deposits activating histone marks (H3K4me3 and H3Ac) and cooperates with E2F1 to enhance transcription [73]. Supporting this dual regulation, ChIP-seq data from Altintas et al. [48] identified ATAD2 as a shared target gene of both AR and E2F1.

### 5.3. P53 and P38-MAPK Mediated Apoptotic Signaling: Attenuated by ATAD2 Expression

Evasion of apoptosis is a hallmark of cancer, and previous studies have shown that ATAD2 plays a critical role in promoting anti-apoptotic activity in cancer cells [54,74,75,76]. Depletion of ATAD2 initiates apoptosis, leading to the cleavage of PARP, a primary apoptotic biomarker in PC [46]. Caron et al. [40] show that ATAD2 levels decrease upon treatment with etoposide, an apoptosis inducer, even before the appearance of cleaved PARP [40]. Moreover, increased ATAD2 expression reduces the caspase-3 activity and apoptotic rate of mechanically stretched retinal pigment epithelial cells [77]. ATAD2 suppression has been shown to boost the expression of pro-apoptotic proteins while reducing the expression of anti-apoptotic proteins in HCC [76]. This was shown through the activation of p53-Bcl-2 family proteins through siATAD2. In the same study, the HCC with mutant p53 shows increased production of phosphorylated p38 (p-p38). Additionally, the study reveals that ATAD2 binds MKK3/6, a regulator of p38 phosphorylation, while ATAD2 suppression encourages MKK3/6 to interact with p38. MKK3/6 phosphorylates p38, activating it and triggering apoptosis [76].

### 5.4. ATAD2 Promotes PI3K/AKT Signaling for Glycometabolism and Cancer Cell Survival

The PI3K-AKT-mTOR signaling cascade regulates key cancer hallmarks, including metabolism, cell cycle, motility, survival, and genomic instability [78]. In BC, ATAD2 has been linked to the pro-survival gene, AKT [65]. Moreover, the BRD-ATAD2 inhibitor AM879 has been reported to induce autophagy via PI3K-AKT-mTOR signaling, suggesting a potential role for ATAD2 in metabolic regulation, although its specific impact on glycometabolism requires further clarification [79].

One of the well-known indicators of malignant tumors is enhanced glycolysis (Warburg effect) with increased expression of hexokinase 2 (HK2) and glucose transporter type 1 (GLUT1) [80,81]. The intense buildup of fluorodeoxyglucose with high total lesion glycolysis is linked to the increased expression of ATAD2. In lung cancer tissues, there is a substantial correlation between the high expression of GLUT1 and HK2 [14]. Further evidence from the same study demonstrates that upregulating ATAD2 causes overexpression of pAKT, GLUT1, and HK2, whereas downregulating ATAD2 causes the opposite effect.

### 5.5. HH Signaling: Activated by Upregulated ATAD2, Which Promotes Carcinogenesis

Aberrant activation of the Hedgehog (HH) signaling pathway contributes to tumor development, and in HCC, ATAD2 depletion reduces the expression of HH pathway proteins [82]. Translationally, the Myc target gene “Miz1” is activated by ATAD2 and is involved in controlling the HH pathway [59]. The overexpression of ATAD2 induces the production of the HH pathway proteins (SHH, SMO, GLI, and PTCH1) in Rb and esophageal squamous cell carcinoma cells (ESCC), which in turn promotes invasion, migration, and proliferation [60,83]. As anticipated, suppressing ATAD2 (either with siATAD2 alone or combined with HH pathway inhibitor, Cyc) results in the opposite effect [60].

### 5.6. Hypoxia Signaling Mediated by HIF1α Stimulates ATAD2 to Promote the Growth of Cancer

HIF1 functions as a master regulator and transcription factor in the hypoxic environment, generally found in solid tumors [84]. Due to cytoplasmic accumulation and translocation into the nucleus, HIF1α combines with HIF1β to create the active HIF1 heterodimeric complex in hypoxic conditions. HIF1α also binds to the target genes’ promoters, increasing the expression of the target genes and triggering carcinogenic pathways [84]. According to two microarray tests, ATAD2 is overexpressed in hypoxic HCC and PC, and was also found to be regulated by the HIF1α-regulated gene [85,86]. The growth and migration of GC cells are further encouraged by the increased expression of ATAD2 [85].

### 5.7. ATAD2 in Epithelial to Mesenchymal Transition (EMT) Pathway

EMT is a pivotal step in cancer progression driven by multiple signaling pathways, endowing cancer cells with invasive properties marked by upregulation of mesenchymal markers (vimentin, N-cadherin, fibronectin, type I collagen, laminin 5, MMPs) and downregulation of epithelial markers (E-cadherin, claudins, occludins, desmoplakin, type IV collagen, laminin 1) [87,88]. Higher expression of ATAD2 helps cancer cells to migrate and invade more readily through the EMT [36]. It has also been reported that ATAD2 knockdown inhibits the metastatic process [61,89]. ATAD2 inhibition reduces the activity of EMT regulators such as snail and slug in CRC and oral squamous cell carcinoma (OSCC) [89]. Yang et al. [90] demonstrated that ATAD2 is indirectly linked to NF-κB signaling by directly targeting the histone methyltransferase NSD2, a key NF-κB chromatin regulator whose overexpression promotes proliferation, survival, and angiogenesis in PC cells [90].

These studies underscore the central role of ATAD2 in multiple malignancies, establishing its suppression as a promising anti-cancer strategy.

## 6. Therapeutic Strategies for ATAD2 Suppression

Targets for small-molecule inhibitors have been focused on two functionally important regions of ATAD2: AAA-ATAD2 and BRD-ATAD2 [36]. Although the AAA+ ATPase domain of ATAD2 has a significant biological function, small molecules designed to target it still require further investigation. This could be due to the high degree of homology with other AAA+ ATPase family members and the unresolved three-dimensional structure of the AAA+ ATPase domain. There are only a few reports on targeting this domain [91,92,93].

The determination of the three-dimensional structure of the drug targets immensely helps structure-based drug design research. Likewise, the X-ray-derived crystal structures of the BRD have helped in the development of novel ATAD2 inhibitors [22,94]. Targeting BRD-ATAD2, however, has been a challenging endeavor in comparison to the other druggable BRD family members because of its distinct binding pocket. Apart from the conserved Tyr1021 and Asn1064 residues, the binding pocket has considerable sequence diversity. The binding cavity of the BRD in ATAD2 is shallow and polar due to the negatively charged loops [95]. The structure is made flexible by the Pro residues at the ZA-BC loops and a particular RVF shelf (WPF in other BRD families). Furthermore, compared to other BRDs, the uneven flexibility of the KAc pocket in BRD-ATAD2 makes drug discovery efforts more difficult [36].

Here, we discuss the various strategies that have been explored to develop inhibitors targeting ATAD2.

**Figure 6:**
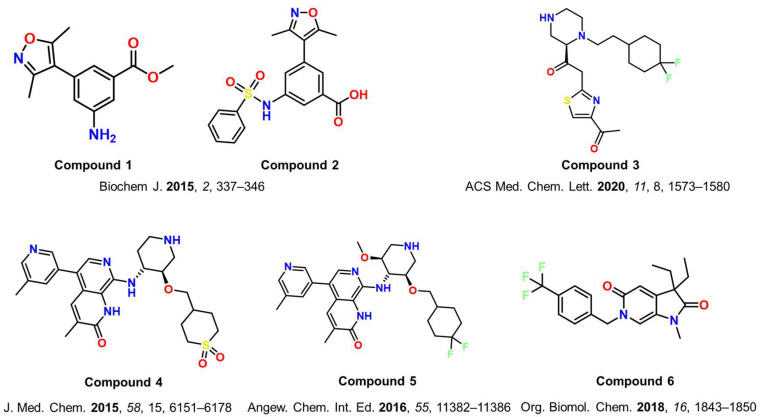
Chemical structures of some of the reported ATAD2 inhibitors discovered through structure-based drug design (compounds **1**–**6**) [38,97,98,99,100].

Through acetyl group mimicking, dimethylisoxazole-based ligands (compounds **1** and **2**, Figure 6) were discovered as putative ATAD2 inhibitors through structure-based methods by Poncet-Montange et al. [38]. MD simulations of reported crystal structures revealed multiple conformations of BRD-ATAD2, reinforcing that the conserved Asn1064 residue adopts distinct conformations [38,96]. This underscores the critical need to consider ATAD2 flexibility in structure-based drug design. Accordingly, MD simulation–based free energy methods provide a robust strategy for discovering novel ATAD2 inhibitors, while induced-fit and ensemble docking approaches offer complementary, lower-cost alternatives.

Another good example of the synergy between computational and experimental work was provided by Dolbois et al. [97]. A moderate-affinity ATAD2 inhibitor (compound **3**, Figure 6) with an IC_50_ value of 15 μM (measured through time-resolved (TR)-FRET assay) was optimized commencing from a hit molecule discovered through pharmacophore modeling. The study also mentions the flexible and adaptive nature of the ZA loop that had a significant effect on the binding of the reported ligands [97]. This study further confirmed that the gatekeeper residues Ile1074, Val1008, and Val1013 can adopt multiple orientations depending on the ligand and even when interacting with the same ligand.

Bamborough et al. [98] discovered naphthyridone-based compounds (compound **4**, Figure 6) that exhibit a sub-nM affinity for BRD-ATAD2 [98]. These compounds, however, were highly hydrophilic and exhibited passive cell permeability, making them less effective. The team subsequently reported another work in which they employed the -CF_2_ group as an isostere for sulfone analogs, retaining the sulfone’s favorable ATAD2 interactions and selectivity over BETs while significantly improving the LogD and membrane permeability. GSK8814 (compound **5**, Figure 6) emerged as the most effective, selective over BET, and cell-permeable chemical probe against BRD-ATAD2 [99]. Later, the same group published a study in which they used conformation restriction using 2-carbon-bridged piperidine (tropane) derivatives starting with compound **5**. The process resulted in the discovery of an ATAD2 inhibitor with high potency (pIC_50_ of 6 against ATAD2 in FRET assay) and having over 250-fold selectivity over the BET BRDs [101]. Another structure-based optimization of a hit led to the discovery of pyrrolidinopyridone scaffolds that inhibited ATAD2 at dose ranges between 100 and 200 μM in Homogeneous Time Resolved Fluorescence (HTRF) assay [100]. Of all the compounds reported in this study, compound **6**, Figure 6, was the best performing compound, having an IC_50_ of 163 µM against ATAD2.

**Figure 7:**
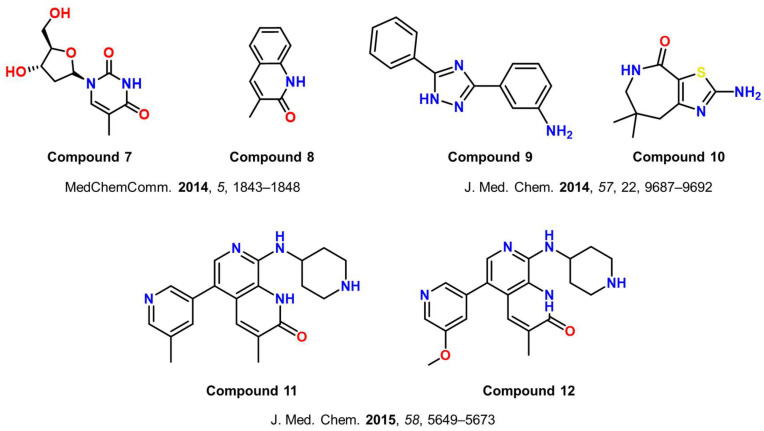
Chemical structures of some of the reported ATAD2 inhibitors discovered through fragment-based drug design (compounds **7**–**12**) [95,102,103].

Using crystallographic fragment screening, Chaikuad et al. [95] initially identified nine novel hits with weak affinity for ATAD2 in 2014. These hits included thymidine (compound **7**, Figure 7) and 3-methylquinoline-2 (1H)-one (compound **8**, Figure 7). The co-crystal structure of compound **7** with BRD-ATAD2 was also reported in the RCSB protein databank (PDB ID: 5A5O). The reported fragments paved the way for the development of potential high-affinity ATAD2 inhibitors starting from these initial chemical moieties. 

**Figure 8:**
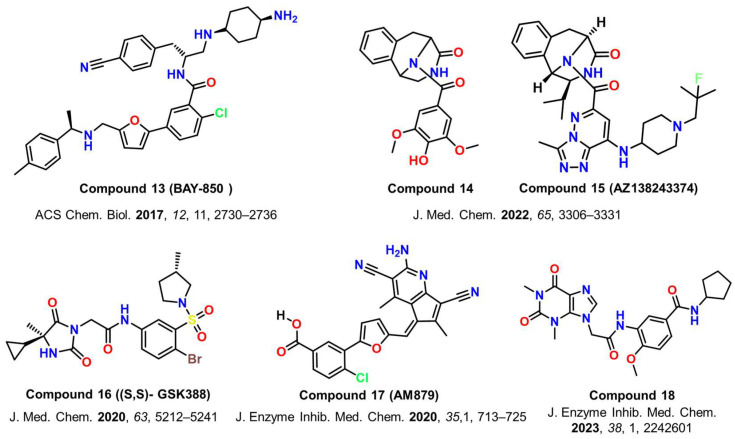
Chemical structures of some of the reported ATAD2 inhibitors discovered through virtual screening or TR-FRET-based high-throughput screening (compounds **13**–**18**) [79,104,106,107,108].

Harner et al. [102] discovered 65 fragments of different chemotypes using the fragment-based approach. Only 12 of these fragments had a K_d_ of less than 1 mM for BRD-ATAD2. The top two compounds from the study, compound **9** (K_d_ of 350 µM) and compound **10** (K_d_ of 500 µM), had the highest binding affinities and are shown in Figure 7 [102]. Later, using the same methodology, Demont et al. [103] identified a variety of quinolinone derivatives (compounds **11** and **12**, Figure 7) as acetyl-lysine mimetics that target BRD-ATAD2, taking inspiration from fragments reported by Chaikuad et al. [95,103]. The protein-bound structure of Compound **12** is available in the Protein Data Bank under PDB ID 5A5R.In a different study, a comprehensive DNA-encoded pool screening led to the development of BAY-850 (compound **13**, Figure 8), a strong and isoform-selective ATAD2 inhibitor with an IC_50_ value of 22 nM. Initial research suggests that BAY-850 may selectively enhance dimerization of ATAD2 bromodomain and inhibit binding to acetylated histones. BAY-850 and related compounds were also investigated for their antiproliferative potential against breast and lung cancer cells. However, a weak correlation was established between biochemical ATAD2 potency and growth inhibition [104]. Later, Guruvaiah et al. [105] found that it inhibited ovarian cancer development and metastasis in both in vitro and in vivo models. They showed that the BAY-850 therapy in cancer cells reduced the expression of centromeric protein (CENP) genes. CENPs govern centromere function, mitosis, and tumor cell growth. The CENPE inhibitor GSK923295, when used in combination therapy with BAY-850, considerably enhanced its tumor-suppressive effects in ovarian cancer cells (PA-1 and SK-OV3) [105].

Winter-Holt et al. [106] performed high-throughput screening through TR-FRET, beginning with 1.8 million samples to find a tetrahydroisoquinoline hit (compound **14**, Figure 8) with an IC_50_ value of 21.2 μM. These derivatives were hydrophilic in nature, which would consequently lead to poor cell permeability. In an attempt to improve the challenges posed, further structural optimization and incorporating weakly basic side chains in the molecule, like (2-fluoro-2-methylpropyl)-4-methylpiperidine, ultimately led to the discovery of AZ138243374 (compound **15**, Figure 8) as the most potent ATAD2 inhibitor in this study, with a pIC_50_ of 8.2 in the TR-FRET assay. A dose-dependent inhibition by AZ13824347 was observed in a colony formation assay against a panel of BC cell lines [106].

Another study based on X-ray crystallography and structural optimization of phenyl sulfonamide derivatives, (S, S)-GSK388 (compound **16**, Figure 8), was reported as an effective and selective ATAD2 inhibitor, having a pIC_50_ of 7.2 in the TR-FRET assay. The reported inhibitor simulates the interactions of KAc histone substrate through critical engagements with conserved Asn and Tyr residues inside the binding site. Remarkably, GSK388 portrayed a >200-fold selectivity for ATAD2 over the BET family [107].

Yao et al. [108] utilized virtual screening to identify AM879 (compound **17**, Figure 8), a new class of BRD-ATAD2 inhibitor. The compound was found to be selective towards BRD-ATAD2, having no inhibitory activity against other BRDs. Despite having a greater inhibitory concentration against ATAD2 (3.56 µM-determined using the TR-FRET assay), AM879 exhibited good antiproliferative activity in BC cells with an IC_50_ value of 2.43 μM and significantly boosted autophagy and apoptosis through PI3K-AKT-mTOR signaling. AM879 was also reported to decrease the expression of c-Myc and ATAD2 [79]. The same group then reported another ATAD2 inhibitor with an IC_50_ = 0.27 µM (compound **18**, Figure 8). Compound **18** effectively reduced c-Myc activation while inducing apoptosis and inhibiting migration in BT-549 cells [108].

Targeting microRNAs (miRs) is another form of cancer therapy that can be employed either as a single agent or combined with conventional treatments [109]. There have been reports of various miRs that downregulate ATAD2’s expression, inhibiting cancer growth and progression [110,111]. miR-372 has been shown to bind to the 3′ UTR section of ATAD2 and inhibit its expression, suppressing oncogenesis in cancers of the liver, ovaries, and kidneys [57,61,112]. In gastric cancer, the downregulation of miR-520f that targets ATAD2 resulted in increased ATAD2 expression and, hence, increased cell proliferation [89]. Next, miR-520a downregulates VEGFA in CRC cells, which lowers ATAD2 expression and prevents angiogenesis [111]. Another long noncoding RNA (lncRNA)-NEAT1_2, is highly upregulated in papillary thyroid carcinoma (PTC) and promotes the expression of ATAD2, which aids in the growth and spread of the tumor [113]. However, by upregulating miR-106b-5p and further downregulating ATAD2, the reduction in NEAT1_2 limits its carcinogenic effects. Higher levels of miR-186 downregulate ATAD2 in retinoblastoma cells and suppress angiogenesis [83].

Despite varying small molecule scaffolds, reported ATAD2 inhibitors share a few fundamental structural principles, explaining their inhibitory action against a similar binding site. First, numerous chemotypes (e.g., dimethylisoxazole derivatives, naphthyridones, pyrrolidinopyridones, and quinolinones) act as acetyl-lysine mimetics, binding to the conserved Asn1064 residue and establishing hydrogen bonds that stabilize the KAc pocket. Second, BRD-ATAD2’s ZA and BC loop flexibility allows chemically diverse scaffolds, such as sulfone analogues, phenyl sulfonamides, and tetrahydroisoquinolines, to accommodate themselves within the shallow, polar binding cavity, highlighting the protein’s adaptability to different ligands. Third, ATAD2’s selectivity and efficacy are frequently boosted by hydrophobic interactions with gatekeeper residues (Val1008, Val1013, Ile1074) or by making use of the distinctive RVF shelf, which distinguishes ATAD2 from other BRDs. For instance, substitution approaches (–CF_2_ vs. sulfone groups in GSK8814) and conformational restriction (tropane derivatives) improved both selectivity and cellular permeability, the latter assessed using an artificial membrane permeability (PAMPA) assay [99]. Finally, functional distinctions between scaffolds appear in cellular contexts: BAY-850 stabilizes ATAD2 dimerization, whereas AM879 has an influence on PI3K-AKT-mTOR signaling and c-Myc production, indicating that scaffold-specific effects modulate downstream biology in addition to core bromodomain recognition. These SAR insights show that, despite different chemotypes, their inhibitory effect is based on acetyl-lysine mimicry and exploitation of ATAD2’s flexible binding site, whereas peripheral changes influence selectivity, cell permeability, and cellular consequences.

## 7. Overview of the Important Amino Acid Residues Involved in the Interaction with Reported Ligands

**Figure 9:**
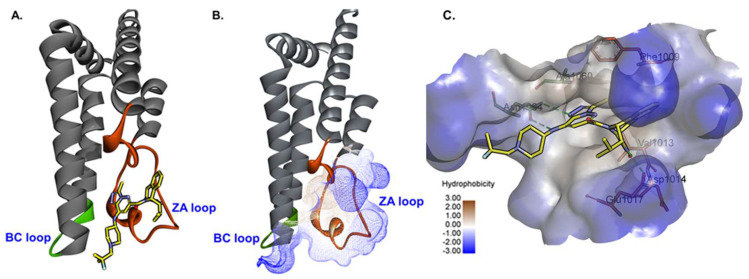
Overview of the ATAD2 binding site. (**A**) The co-crystal structure of the inhibitor bound to the target protein (PDB ID: 7Q6T). The ZA loop is highlighted in orange, and the BC loop is shown in green. (**B**) The binding site of BRD-ATAD2 is shown in surface representation, depicted with mesh style, highlighting the hydrophobicity of the pocket. (**C**) The binding site is shown with some critical amino acid residues with the reported inhibitor as reported in PDB ID: 7Q6T. Green dashed lines represent hydrogen bonds.

**Figure 10:**
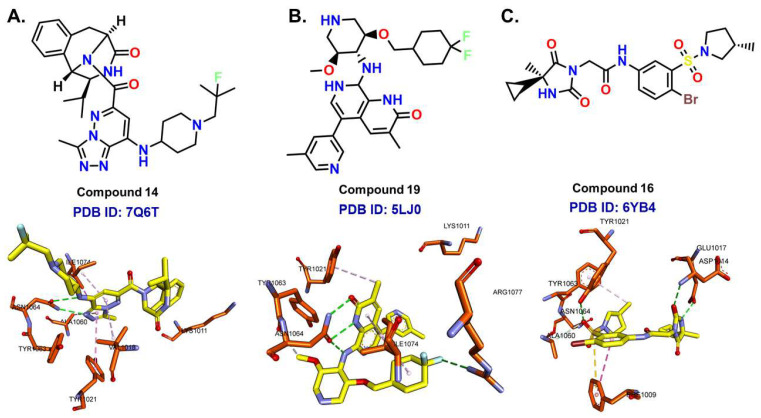
(**A**–**C**) depict the chemical structures of ligands and their interactions with key binding site residues of ATAD2 in their respective crystallographic poses. Green dashed lines represent hydrogen bonds; cyan dashed lines indicate halogen bonds and pink dashed lines denote other critical interactions such as π-alkyl and π-π interactions.

X-ray crystallography delivers high-resolution structural insights into protein-ligand interactions, capturing a detailed yet static view of binding site interactions. The first crystal structure of the ATAD2 bromodomain was reported by Filippakopoulos et al. [114]. Though valuable, crystallographic data alone may not fully capture the dynamic nature of protein-ligand interactions [115]. As highlighted in several reports in the previous section, understanding the flexibility of the target BRD-ATAD2 (Figure 9) is crucial for future structure-driven molecular modeling studies. Therefore, we went on to perform MD simulations and molecular mechanics with generalized Born and surface area solvation (MM/GBSA) calculations, incorporating computational alanine scanning to identify key binding site residues. This approach can help us understand the dynamics of protein-ligand binding, as well as the role of binding site amino acid residues in ligand binding in a simulated environment. We selected three ATAD2-inhibitor complexes from the RCSB protein data bank (PDB): 7Q6T (resolution: 2.05 Å), 5LJ0 (resolution: 1.82 Å), and 6YB4 (resolution:1.85 Å). These structures were chosen due to the distinct chemical scaffolds of their inhibitors (Figure 10). Each complex underwent 250 ns MD simulations in duplicates, followed by MM/GBSA calculations on the last 150 ns. Computational alanine scanning was performed by substituting binding site residues within 5 Å of the inhibitor with alanine, evaluating their contributions to binding affinity (ΔΔH = ΔH_mutated_ − ΔH_normal_). In this context, a highly positive ΔΔH value indicates that the specific residue plays a crucial role in ligand binding. Proline and glycine residues were excluded from mutations due to their structural roles, and alanine residues were replaced with glycine where necessary using the gmx_MMPBSA tool. The detailed protocol of the simulation and MM/GBSA calculations is provided in the Supplementary Information.

For the 7Q6T complex, the most significant contributor to binding was Ile1074 (ΔΔH_total_ = 3.08 kcal/mol), located in the hydrophilic pocket. Ile1074 formed a π-σ interaction with the triazolo-pyridazine moiety of the inhibitor in the crystal pose (Figure 10A). This interaction was further supported by alanine scanning calculations, which identified Ile1074 as a key contributor to the total energy difference, primarily due to van der Waals interactions (ΔΔH_vdW_ = 3.17 kcal/mol). Asn1064, a highly conserved BRD residue, also played a major role (ΔΔH_total_ = 2.24 kcal/mol), acting as both a hydrogen bond donor and acceptor through its amide side chain in the crystal pose (Figure 10A). The mutation of Asn1064 to Ala significantly changed the electrostatic contributions to the binding free energy (ΔΔH_ele_ = 3.62 kcal/mol). Additionally, Tyr1063 (ΔΔH_total_ = 2.07 kcal/mol), a ZA loop residue, engaged in van der Waals interactions, as indicated by its ΔΔH_vdW_ contribution. The results are provided in Table 1.

In the 5LJO complex, Asn1064 had the highest impact (ΔΔH_total_ = 4.42 kcal/mol), forming hydrogen bonds (ΔΔH_ele_ = 9.3 kcal/mol) through its amide side chain in its crystal pose (Figure 10B) (Table 2). Tyr1063 (ΔΔG = 2.83 kcal/mol) again exhibited significant van der Waals interactions (ΔΔH_vdW_ = 3.02 kcal/mol). Arg1077 contributed through hydrogen bonding with the fluorine atom of the inhibitor, leading to a notable change in ΔΔH_ele_ upon mutation (ΔΔH_ele_ = 1.59 kcal/mol) (Figure 10B). Interestingly, Lys1011 was found to be a major contributor to electrostatic interactions (ΔΔH_ele_ = 2.11 kcal/mol).

For the 6YB4 complex, the highest contributing residue was Tyr1021 (ΔΔH_total_ = 4.36 kcal/mol), with a substantial impact on ΔΔH_vdW_ (ΔΔH_vdW_ = 4.45 kcal/mol) upon mutation (Table 3). Phe1009 also played a crucial role, likely forming van der Waals interactions with the sulfur atom of the inhibitor, as reflected in its ΔΔH_vdW_ value (Figure 10C). Mutation of Glu1017 and Asp1014 to Ala resulted in the highest changes for ΔΔH_ele_ (ΔΔH_ele_ = 6.02 kcal/mol and ΔΔH_ele_ = 4.94 kcal/mol, respectively). They were found to be forming hydrogen bonds with the inhibitor (Figure 10C). Additionally, the mutation of Ala1060 to Gly also resulted in a significant ΔΔH_ele_ (3.25 kcal/mol). Ala1060 forms carbon-hydrogen bonds with the Br and sulfone moieties of the inhibitor (Figure 10C).

Collectively, our analysis identified key ATAD2 residues involved in inhibitor binding: Arg1007, Val1008, Phe1009, Lys1011, Val1013, Asp1014, Glu1017, Val1018, Tyr1021, Ala1060, Tyr1063, Asn1064, Ile1074, and Arg1077. Consistent with previous reports, these residues are critical for ATAD2 inhibition, with Arg1007, Val1008, Phe1009, Lys1011, Val1013, Asp1014, Glu1017, Val1018, and Tyr1021 located on the ZA loop, and Tyr1063 and Asn1064 situated on the BC loop [15,100,104,106,107,108,116]. These findings refine our understanding of ATAD2-ligand interactions beyond crystallographic data, offering a dynamic perspective on binding site residues under physiological conditions. The integration of MD simulations, MM/GBSA calculations, and computational alanine scanning provides a comprehensive evaluation of residue contributions, aiding future inhibitor design.

## 8. Role of ATAD2 in Cancer Drug Resistance

Dysregulation of histone-modifying enzymes, including HDACs, histone acetyltransferases (HATs), and histone methyltransferases, contributes to oncogene activation and tumor suppressor silencing, processes that are closely linked to the development of resistance to chemotherapy and targeted therapies. Tumor hypoxia further exacerbates this resistance by promoting global chromatin compaction through increased HDAC activity, resulting in transcriptional repression and decreased drug sensitivity. ATAD2 counteracts HDAC2-mediated deacetylation, maintaining histone hyperacetylation and facilitating oncogenic transcription. Since histone acetylation regulates transcriptional programs that determine cellular responses to therapy, cancer cells exploit epigenetic mechanisms, including ATAD2-mediated chromatin modulation, to evade cytotoxic effects [25,117,118,119,120,121]. Notably, under severe hypoxia, ATAD2 expression decreases, leading to reduced H3 acetylation, chromatin condensation, and S-phase cell cycle arrest, which confers resistance to topoisomerase inhibitors, platinum-based drugs, and taxanes [122]. Conversely, forced ATAD2 expression can restore cell cycle progression and enhance chemosensitivity in hypoxic cancer cells [122,123].

ATAD2 has been found to be a paclitaxel resistance marker in GC with peritoneal metastases [124]. ATAD2 amplifies the transcription of the oncogenic ubiquitin E3 ligase TRIM25 and establishes a positive feedback circuit (ATAD2-E2Fs-TRIM25) that promotes CRC progression and therapy resistance [55]. Furthermore, Lie et al. [125] found that ATAD2 is engaged in the CRNDE-mediated miR-126-5p/ATAD2 axis, in which the long noncoding RNA CRNDE suppresses miR-126-5p, increasing ATAD2 expression and elevating paclitaxel resistance in CRC cells [125]. According to Wang et al. [126], a microRNA called miR-200b-5p has been found to directly target ATAD2, leading to the suppression of PI3K/AKT signaling. This results in the reduction in ovarian cancer cell proliferation and the promotion of apoptosis. Furthermore, in the same study, it was noted that BAY-850, an inhibitor of ATAD2, dramatically reduced the amount of ATAD2 protein. This also resulted in a reduction in the proliferative capacity of cancer cells while improving the rate of apoptosis [126]. Platinum-based chemotherapy resistance in OC has also been linked to ATAD2 and its impact on signaling pathways, as demonstrated by Ge et al. [127], underscoring ATAD2 as a crucial chemoresistance regulator [127]. It has also been shown that ATAD2 increases the resistance of GBM to radiation therapy by transcriptionally regulating PLK4, an essential mitotic kinase [128]. ATAD2 is essential for regulating gemcitabine and radiation treatment resistance in pancreatic ductal adenocarcinoma (PDAC). The main conclusions from this study indicate that ATAD2 suppression causes apoptosis and autophagy, downregulates DNA repair proteins (pChk1 and pChk2), increases DNA damage, and improves radiosensitivity [58]. These observations underscore ATAD2 as a prospective target for therapy to sensitize PDAC cells to treatment.

Given the evidence linking ATAD2 to paclitaxel resistance across multiple cancers, particular attention should be given to assessing these compounds in paclitaxel-resistant models, with in vivo validation using resistant xenografts to support clinical translation. Furthermore, ATAD2 inhibitors can also be employed in combination with gemcitabine to validate the observations of Dutta et al. [58], offering further details on how these strategies improve the response of PC to chemotherapy. Beyond chemoresistance, ATAD2 contributes to tumor progression and immune evasion; for instance, in papillary thyroid cancer (PTC), it promotes oncogenic behaviors by activating the PI3K-AKT pathway and regulating the G1/S cell cycle transition [129]. ATAD2 also facilitates immune suppression through interactions with inhibitory immune checkpoints and tumor-infiltrating cells. This immune dysfunction represents a critical barrier to effective immunotherapy, underscoring ATAD2 as a key regulator of both drug resistance and immune evasion in cancer.

## 9. Current Challenges and Future Directions

Given the significant function of ATAD2 in numerous cancers, the hunt for small-molecule inhibitors that target ATAD2 has been an increasingly prevalent subject in oncological research. Initially, the results of computational studies indicated that ATAD2’s expected druggability was poor; however, subsequent research on this enzyme has shown it to be a pharmacologically modulable target. The crystal structures of BRD-ATAD2 have proven useful for drug discovery and computational druggability assessment [94,130]. Compared with BRD4’s hydrophobic WPF shelf (Trp–Pro–Phe), the ATAD2 bromodomain features an RVF shelf (Arg–Val–Phe) and a more polar, shallow binding pocket; increased flexibility arises primarily from the ZA/BC loops rather than the shelf itself [15]. It has a hydrophilic binding site due to another unique Arg1077 in place of the BRD4 BD1 residue Met149. Additionally, ATAD2’s ZA loop is two residues shorter than BRD4’s, which increases the KAc site’s hydrophilicity and relative solvent exposure. Likewise, the AAA+ ATPase domain encounters difficulties searching for new drugs due to the absence of a detailed crystal structure for this domain and its strong resemblance to other members of the AAA+ ATPase family [100]. Even though there has been significant progress in drug discovery studies that target ATAD2, there are still many obstacles to overcome before these drugs are put through clinical trials [106]. Small-molecule inhibitors targeting BRD-ATAD2, which have been explored over the course of the past decade, such as GSK8814, AZ13824374, AM879, BAY-850, and others, were examined in this review. The majority of these inhibitors have demonstrated limited effectiveness in inhibiting cancer cell proliferation and other important pathways driving tumor growth despite their promising biochemical action. In addition, we investigated ATAD2’s structural characteristics and function in multiple oncogenic signaling pathways, including Rb/E2F-cMyc, PI3K/AKT/mTOR, HH signaling, and steroid hormone signaling. Despite rising evidence of ATAD2’s role in carcinogenesis, further research is needed to determine its precise actions across different cancer types. Future research should prioritize the development of ATAD2-specific inhibitors and evaluate their therapeutic potential in overcoming drug resistance. These inhibitors should be tested in therapy-resistant cancer cell lines, both as single agents and in combination with conventional chemotherapy or radiotherapy. Assessing their effects on resistant cancer phenotypes will provide critical insights into whether ATAD2 inhibition can restore drug sensitivity and advance these compounds toward clinical application. An extensive pharmacokinetic investigation through in vitro and in vivo experiments is required to determine their metabolic stability, toxicity, and clearance. Furthermore, cytotoxicity tests on non-malignant cells will be critical in assessing the selectivity and safety profile of these inhibitors. Prospective studies should also focus on improving the potency, selectivity, and drug-like features of ATAD2 inhibitors in order to improve clinical translation. Expanding structure-activity relationship (SAR) investigations, using computational modeling for rational drug design, and investigating new chemical scaffolds may aid in the identification of more effective ATAD2-targeting drugs. Expanding on the aforementioned information would enhance the comprehension of ATAD2’s role in cancer progression and open up new avenues for developing novel anticancer therapeutics.

## 10. Conclusions

In this review, we provided a comprehensive examination of ATAD2, beginning with its production, regulation, structural features, and functional characterization. We detailed its biological activities with a particular focus on its role in cancer, highlighting how ATAD2 functions as a transcriptional co-regulator through interactions with acetylated histones and key transcription factors. Its interplay with E2F1, MYC, and hormone receptors such as ER and AR underscores its central role in promoting cell cycle progression, DNA replication, metabolic adaptation, and hormone-driven tumorigenesis. Furthermore, ATAD2 influences DNA replication and repair pathways, suggesting a potential contribution to therapeutic resistance, particularly in the context of chemotherapeutic stress. We then discussed current therapeutic strategies targeting ATAD2, emphasizing small-molecule inhibitor development against both the AAA+ ATPase and BRD regions. Structural studies, along with our MD simulations and alanine scanning calculations using MM/GBSA-derived binding free energies from MD trajectories, identified key hotspot residues from both ZA and BC loops, revealing opportunities to exploit loop dynamics and scaffold-specific interactions for selective inhibitor design. Additionally, regulation of ATAD2 by microRNAs highlights the complex epigenetic and signaling networks in which ATAD2 operates, offering further avenues for therapeutic intervention. Emerging studies also point to ATAD2’s role in mediating drug resistance, particularly in paclitaxel- and gemcitabine-resistant models, reinforcing its potential as a target to enhance chemotherapeutic efficacy.

While challenges remain, including the high homology of the AAA+ domain and the need for in vivo validation of inhibitors, our review provides a roadmap for integrating structural insights, mechanistic studies, and rational drug design to optimize ATAD2-targeted therapies. Overall, we hope this review serves as a valuable reference for researchers, providing a foundation for future studies aimed at exploring ATAD2’s full therapeutic potential in cancer. By highlighting ATAD2’s central roles in oncogenic signaling, chromatin remodeling, and drug resistance, this review emphasizes its potential as a critical therapeutic target, aiming to motivate medicinal chemists, pharmacologists, cancer biologists, and other researchers to advance ATAD2-focused drug discovery and translational studies.

## Figures and Tables

**Table 1:** Results of the alanine scanning calculations for the complex with PDB ID 7Q6T. The alanine scanning was performed using MM/GBSA-derived binding free energies calculated from MD-simulated trajectories.

PDB ID: 7Q6T			
Residues	ΔΔH (vdW)	ΔΔH (ele.)	ΔΔH (Total)
ZA loop; ARG1007:ALA	0.83 ± 0.17	−1.86 ± 0.11	0.67 ± 0.14
ZA loop; VAL1008:ALA	0.76 ± 0.00	0.04 ± 0.02	0.68 ± 0.02
ZA loop; PHE1009:ALA	0.90 ± 0.03	0.20 ± 0.02	0.91 ± 0.03
ZA loop; THR1010:ALA	−0.02 ± 0.01	1.25 ± 0.07	0.22 ± 0.01
ZA loop; LYS1011:ALA	1.10 ± 0.06	−0.99 ± 0.12	1.08 ± 0.07
ZA loop; VAL1013:ALA	0.68 ± 0.01	−0.33 ± 0.03	0.85 ± 0.01
ZA loop; ASP1014:ALA	0.49 ± 0.03	−0.03 ± 0.11	−0.03 ± 0.02
ZA loop; GLU1017:ALA	0.71 ± 0.02	0.95 ± 0.13	−0.12 ± 0.01
ZA loop; VAL1018:ALA	1.32 ± 0.01	−0.36 ± 0.05	1.17 ± 0.01
ZA loop; ASP1020:ALA	0.13 ± 0.10	−1.24 ± 0.42	−0.23 ± 0.11
ZA loop; TYR1021:ALA	1.50 ± 0.04	0.73 ± 0.03	1.39 ± 0.03
ALA1060:GLY	0.15 ± 0.01	0.99 ± 0.05	0.33 ± 0.02
BC loop; TYR1063:ALA	2.55 ± 0.32	−0.14 ± 0.02	2.07 ± 0.32
BC loop; ASN1064:ALA	1.32 ± 0.03	3.62 ± 0.15	2.24 ± 0.06
ASP1071:ALA	0.47 ± 0.04	0.33 ± 0.24	−0.39 ± 0.02
ILE1074:ALA	3.17 ± 0.03	0.36 ± 0.02	3.08 ± 0.03

**Table 2:** Results of the alanine scanning calculations for the complex with PDB ID 5LJO. The alanine scanning was performed using MM/GBSA-derived binding free energies calculated from MD-simulated trajectories.

PDB ID: 5LJ0			
Residues	ΔΔH (vdW)	ΔΔH (ele.)	ΔΔH (Total)
ZA loop; VAL1008:ALA	0.92 ± 0.09	−0.41 ± 0.04	0.82 ± 0.58
ZA loop; PHE1009:ALA	0.94 ± 0.01	−0.09 ± 0.01	0.69 ± 0.27
ZA loop; LYS1011:ALA	0.27 ± 0.04	2.11 ± 1.44	0.66 ± 0.79
ZA loop; VAL1013:ALA	0.43 ± 0.16	−0.60 ± 0.12	0.23 ± 0.37
ZA loop; ASP1014:ALA	0.19 ± 0.14	−0.51 ± 0.32	0.01 ± 0.20
ZA loop; GLU1017:ALA	0.60 ± 0.24	−0.11 ± 1.18	0.16 ± 0.37
ZA loop; VAL1018:ALA	0.60 ± 0.33	−0.22 ± 0.03	0.42 ± 0.43
ZA loop; TYR1021:ALA	0.95 ± 0.14	0.48 ± 0.11	0.78 ± 0.42
ALA1060:ALA	0.14 ± 0.00	1.84 ± 0.03	0.48 ± 0.18
BC loop; TYR1063:ALA	3.02 ± 0.06	−0.09 ± 0.23	2.83 ± 0.58
BC loop; ASN1064:ALA	−0.50 ± 0.09	9.30 ± 0.05	4.42 ± 0.94
BC loop; ASP1066:ALA	0.19 ± 0.01	2.45 ± 0.08	−0.37 ± 0.27
ASP1071:ALA	0.13 ± 0.01	3.21 ± 0.53	−0.24 ± 0.29
LEU1073:ALA	0.51 ± 0.06	0.11 ± 0.01	0.52 ± 0.34
ILE1074:ALA	2.47 ± 0.05	−0.13 ± 0.06	2.52 ± 0.58
ARG1077:ALA	0.81 ± 0.07	1.59 ± 0.22	0.86 ± 0.60

**Table 3:** Results of the alanine scanning calculations for the complex with PDB ID 6YB4. The alanine scanning was performed using MM/GBSA-derived binding free energies calculated from MD-simulated trajectories.

PDB ID: 6YB4			
Residues	ΔΔH (vdW)	ΔΔH (ele.)	ΔΔH (Total)
ZA loop; ARG1007:ALA	0.05 ± 0.01	0.34 ± 0.01	0.02 ± 0.01
ZA loop; VAL1008:ALA	0.54 ± 0.07	−0.53 ± 0.39	0.37 ± 0.15
ZA loop; PHE1009:ALA	2.14 ± 0.15	0.08 ± 0.02	1.96 ± 0.17
ZA loop; THR1010:ALA	0.01 ± 0.01	−0.06 ± 0.16	0.13 ± 0.01
ZA loop; LYS1011:ALA	1.13 ± 0.06	0.17 ± 0.62	0.99 ± 0.24
ZA loop; VAL1013:ALA	0.60 ± 0.24	−0.66 ± 0.12	0.67 ± 0.22
ZA loop; ASP1014:ALA	−0.06 ± 0.29	4.94 ± 3.99	0.96 ± 0.64
ZA loop; GLU1017:ALA	−0.21 ± 0.23	6.02 ± 5.65	1.04 ± 1.03
ZA loop; VAL1018:ALA	0.23 ± 0.21	−0.09 ± 0.04	0.20 ± 0.19
ZA loop; TYR1021:ALA	4.45 ± 1.03	0.21 ± 0.39	4.36 ± 1.14
ILE1056:ALA	0.26 ± 0.02	−0.66 ± 0.05	0.15 ± 0.03
ALA1060:GLY	0.22 ± 0.04	3.25 ± 0.16	1.17 ± 0.05
BC loop; TYR1063:ALA	2.02 ± 0.21	−0.41 ± 0.04	1.65 ± 0.21
BC loop; ASN1064:ALA	1.30 ± 0.06	1.15 ± 0.05	1.64 ± 0.17
ILE1074:ALA	1.11 ± 0.04	−0.67 ± 0.27	0.81 ± 0.14
ALA1078:GLY	0.05 ± 0.00	1.38 ± 0.14	0.24 ± 0.00

## Supplementary Materials

The following supporting information can be downloaded at: https://www.mdpi.com/article/10.3390/cancers17203337/s1, References [131,132,133,134,135] are cited in the supplementary materials.

## Abbreviations

The following abbreviations are used in this manuscript:

ATAD2	ATPase family AAA domain-containing protein 2
BRD	bromodomain
ANCCA	AAA+ nuclear coregulator cancer-associated
CTA	cancer-testis antigen
ES	embryonic stem cells
Rb	retinoblastoma
ERα	estrogen receptor alpha
ATP	adenosine Triphosphate
ADP	adenosine diphosphate
DNA	deoxyribonucleic acid
MD	molecular dynamics
AIB1	amplified in breast cancer 1
RAC3	receptor-associated coactivator 3
TRAM1	translocation-associated membrane protein 1
E2	estradiol
ORF	open reading frame
cAMP	cyclic adenosine monophosphate
CBP	CREB-binding protein
p300	EP300 or E1A binding protein p300
E2F	early region 2 binding factor
c-Myc	cellular myelocytomatosis
AKT	protein kinase B
HIF1α	hypoxia inducible factor 1 subunit alpha
GBM	glioblastoma
MAPK	mitogen-activated protein kinase
Asn	asparagine
kDa	kilodaltons
E2F1	early region 2 binding factor transcription factor 1
VCP	valosin-containing protein
Hsp104	heat shock protein 104
NSF	N-ethylmaleimide-sensitive factor
Arg	arginine
Abo1	ATP-binding cassette subfamily F member 1
Pro	proline
Val	valine
MCM7	minichromosome maintenance complex 7
SRC-3	steroid receptor coactivator-3
RNA	ribonucleic acid
CDK	cyclin-dependent kinases
NCOA3	nuclear receptor coactivator
B-MYB	B-Myeloid myeloblastosis
OS	osteosarcoma
TNBC	triple-negative breast cancer cells
Top2A	DNA topoisomerase II alpha
DLGAP5	discs large-associated protein 5
Bub1	budding uninhibited by benzimidazole 1
MCM10	minichromosome maintenance protein 10
Kif	kinesin family
NOL1	nucleolar protein 1
GPAT	glycerol-3-phosphate acyltransferase
HSP60	heat shock protein 60
CAD	carbamoyl-phosphate synthetase 2, aspartate transcarbamylase, and dihydroorotase
CDC25A	cell division cycle 25A
AIM1	absent in melanoma 1
CCNE1	cyclin E1
ATR	ataxia telangiectasia and Rad3-related protein
MLL1	mixed lineage leukemia protein-1
IRS-2	insulin receptor substrate 2
SNK	serum-inducible protein kinase
AR	androgen receptor
EZH2	enhancer of zeste homolog 2
PARP	poly(ADP-ribose) polymerase
PC	prostate cancer
HCC	hepatocellular carcinoma
Bcl-2	B-cell leukemia/lymphoma 2 protein
MKK3/6	mitogen-activated protein kinase 3/6
PI3K	phosphatidylinositol 3-kinases
mTOR	mammalian target of rapamycin
pAKT	phosphorylated protein kinase B
Miz1	c-Myc-interacting zinc finger protein-1
ESCC	esophageal squamous cell carcinoma
SHH	sonic hedgehog protein
SMO	smoothened
GLUT1	glucose transporter type 1
HK2	hexokinase 2
GLI	glioma-associated oncogene
EMT	epithelial to mesenchymal transition
MMPs	matrix metalloproteases
PTCH1	protein patched homolog 1
CRC	colorectal cancer
OSCC	oral squamous cell carcinoma
NF-κB	nuclear factor kappa-light-chain-enhancer of activated B cells
NSD2	nuclear receptor binding SET domain protein 2
KAc	acetyl-lysine
IC50	half-maximal inhibitory concentration
Ile	isoleucine
BET	bromodomain and extraterminal
RCSB	research collaboratory for structural bioinformatics
PDB	protein data bank
Kd	equilibrium dissociation constant
TR-FRET	time-resolved fluorescence resonance energy transfer
NEAT1_2	nuclear enriched abundant transcript 1, isoform 2
MM/GBSA	molecular mechanics with generalized Born and surface area solvation
miRs	microRNAs
UTR	untranslated region
CENP	centromeric protein
Tyr	tyrosine
Phe	phenylalanine
Glu	glutamic acid
Asp	aspartic acid
Ala	alanine
Gly	glycine
Br	bromine
GC	gastric cancer
TRIM25	tripartite motif-containing protein 25
miR-126-5p	microRNA-126-5p
OC	ovarian cancer
pChk	phosphorylated checkpoint kinase
PTC	papillary thyroid carcinoma
CD8Tex	cluster of differentiation 8 T cell exhaustion
Trp	tryptophan
Met	methionine
SAR	structure-activity relationship
HATs	histone acetyltransferases
HDACs	histone deacetylases
CRNDE	colorectal neoplasia differentially expressed
PDAC	pancreatic ductal adenocarcinoma
