# Supplementary material for: ATAD2 as a Cancer Target: Insights into Its Structure, Functions, Mechanisms, and Drug Development
Source: Cancers (Basel). 2025 Oct 16;17(20):3337. doi: 10.3390/cancers17203337 (PMC12563300; doi:10.3390/cancers17203337)
Supplement: Supplementary file 1 [file cancers-17-03337-s001.zip › cancers-3872405-supplementary.pdf]

Molecular dynamics (MD) simulation protocols.

Protein-ligand complexes were acquired from the RCSB Protein Data Bank. Protein structures were refined using Swiss PDB-Viewer 4.1 [131], which removed water and ions, repaired missing atoms, and performed GROMOS forcefield-based energy minimization. The crystallographic ligand poses served as the starting geometries for MD simulations, which were carried out using GROMACS 2024.3 [132] with the CHARMM36 force field [133] for the protein and the CHARMM General Force Field (CGenFF) [134] for ligands. The complexes then underwent solvation, neutralization (with Na^+ and Cl^-) and minimization (5000 steepest descents and 10,000 conjugate gradient steps) in a triclinic box. NVT equilibration was performed for 50 ps to bring the system temperature to 300 K, followed by 1 ns NPT equilibration at 300 K and 1 atm. A 250 ns production run was carried out using particle mesh Ewald (PME) electrostatics and a 1.2 nm van der Waals cutoff. Two replicas were run at varying random velocities, and the results were averaged.

Binding free energy calculations.

Binding free energies were estimated using molecular mechanics with the generalized Born and surface area solvation (MM/GBSA) method and the gmx_MMPBSA tool [135], which included van der Waals (ΔH_{vdW}), electrostatic (DH_{ele}), and solvation energy (ΔH_{solv}) components. The polar solvation energy (ΔH_{polar}) was estimated using GB-OBC2 (igb = 5) with dielectric constants of 1 (solute) and 78.5 (solvent). The non-polar solvation energy ($\Delta H_{non\text{-}polar}$) was computed using the LCPO algorithm ($\Delta E_{non\text{-}polar} = 0.0072 * \Delta SASA$, with a 1.4 Å probe radius).

The binding free energies (ΔH_{total}) of the ligands with the protein were determined using the following formula:

$$\Delta H_{total} = \Delta H_{gas} + \Delta H_{solv}$$

$$\text{where, } \Delta H_{gas} = \Delta H_{vdw} + \Delta H_{ele}$$

$$\text{and } \Delta H_{solv} = \Delta H_{polar} + \Delta H_{non\text{-}polar}$$

Computational alanine scanning calculations.

The alanine scanning calculations were derived from the MD simulation trajectory using the same snapshots utilized for the MM/GBSA calculations. By altering the normal trajectory coordinates, truncating the side chains of modified residues at the Cγ position, and replacing them with hydrogen atoms, alanine mutant structures were produced. The topology files have been updated to incorporate the alanine residue parameters for the changed residues. The binding free energy differences between the mutant and normal complexes were then calculated using the gmx_MMPBSA ($\Delta\Delta H = \Delta H_{mutant} - \Delta H_{normal}$). As stated in the main text, a higher positive value of $\Delta\Delta H$ indicates that the residue contributes more to the ligand binding.

References

131. Guex, N.; Peitsch, M. C. SWISS-MODEL and the Swiss-Pdb Viewer: An Environment for Comparative Protein Modeling. *Electrophoresis* **1997**, *18* (15), 2714–2723. https://doi.org/10.1002/elps.1150181505.

132. Abraham, M. J.; Murtola, T.; Schulz, R.; Páll, S.; Smith, J. C.; Hess, B.; Lindahl, E. GROMACS: High Performance Molecular Simulations through Multi-Level Parallelism from Laptops to Supercomputers. *SoftwareX* **2015**, *1–2*, 19–25. https://doi.org/10.1016/j.softx.2015.06.001.

133. Huang, J.; MacKerell, A. D. CHARMM36 All-Atom Additive Protein Force Field: Validation Based on Comparison to NMR Data. *J Comput Chem* **2013**, *34* (25), 2135–2145. https://doi.org/10.1002/jcc.23354.

134. Vanommeslaeghe, K.; Hatcher, E.; Acharya, C.; Kundu, S.; Zhong, S.; Shim, J.; Darian, E.; Guvench, O.; Lopes, P.; Vorobyov, I.; Mackerell, A. D. CHARMM General Force Field: A Force Field for Drug-like Molecules Compatible with the CHARMM All-atom Additive Biological Force Fields. *J Comput Chem* **2010**, *31* (4), 671–690. https://doi.org/10.1002/jcc.21367.

135. Valdés-Tresanco, M. S.; Valdés-Tresanco, M. E.; Valiente, P. A.; Moreno, E. Gmx_MMPBSA: A New Tool to Perform End-State Free Energy Calculations with GROMACS. *J Chem Theory Comput* **2021**, *17* (10), 6281–6291. https://doi.org/10.1021/acs.jctc.1c00645.
